# Comparison and interobserver reliability of three different methods for measuring the amount of the tibial tuberosity advancement in the preoperative planning of TTAT

**DOI:** 10.1371/journal.pone.0289259

**Published:** 2023-11-09

**Authors:** Giovanni Della Valle, Federica Aragosa, Chiara Caterino, Alfonso Piscitelli, Cristina Di Palma, Francesco Lamagna, Gerardo Fatone

**Affiliations:** 1 Department of Veterinary Medicine and Animal Production, University of Naples "Federico II”, Naples, Italy; 2 Department of Agricultural Sciences, University of Naples "Federico II", Portici, Italy; Sheikh Hasina National Institute of Burn & Plastic Surgery, BANGLADESH

## Abstract

**Introduction:**

The goal of preoperative planning techniques for advancement of the tibial tuberosity is to determine the amount of advancement required to achieve a postoperative patellar tendon angle of 90° and to select the optimal wedge size to achieve this target.

**Material and methods:**

Three radiographic methods for determining the advancement distance for the tibial tuberosity were evaluated for comparability and interobserver reliability. Among the methods developed, we decided to include the common tangent method, the tibial anatomy-based method, and the Bielecki method. For all techniques, radiographs were taken in mediolateral projection with the knee joint flexed at 135°. Three observers with different levels of experience independently evaluated and scored the degree of osteoarthritis for each stifle, as previously described, and performed measurements of the amount of advancement of the tibial tuberosity on 33 stifles using common tangent method, tibial anatomy-based method, and Bielecki method.

**Results:**

According to the results, the overall score for osteoarthritis in the mediolateral view was influenced by the experience of the observers, which contradicts the results from a previous study. Regarding the measurement methods used to assess advancement, poor interobserver reliability was found for common tangent method and Bielecki method, while only a slightly moderate interobserver agreement was found for tibial anatomy-based method.

**Discussion:**

These results are inconsistent with data collected by Bielecki and colleagues. Moreover, measurements from common tangent method and tibial anatomy-based method were overlapping, as confirmed previously. Conversely, Bielecki method showed no agreement with the other methods included in the present study, with a significantly higher mean rank, probably due to its correction formula. Based on the results of the present study, tibial anatomy-based method has better interobserver reliability and is easier to perform according to the observers.

## Introduction

Tibial tuberosity advancement techniques (TTAT) are surgical procedures that aim to lead the patellar tendon angle (PTA) to 90° in dogs with cranial cruciate ligament disease (CCLD) to counteract stifle instability [1]. In literature, different preoperative measurement methods have been proposed to calculate the right advancement amount. The reliability, clinical efficacy, and comparison of some of them have been evaluated in several studies [2–4]. These previous studies suggest that the measurement method may influence the value of the desired advancement, showing a theoretical discrepancy between the preoperatively calculated PTA and the postoperative one [2–4]. There is widespread agreement in the literature that a PTA of 90° ± 5° may be sufficient to adequately neutralize tibiofemoral shear forces in a dog with CCLD, but suboptimal postoperative PTA may result in residual instability despite TTAT [3, 5–7]. The latter condition may explain the meniscal tear rates of 5% after TTA and as high as 28% without meniscal release [8–10]. Although this concept is widely accepted in dogs, it is largely based on a two-dimensional mathematical model of the human knee. Its validity in dogs has been undermined to some extent by the findings of Apelt et al. [6], suggesting that a biomechanically relevant "crossover point" might not exist in dogs [6].

Radiological methods described to assess the proper amount of advancement for the tibial tuberosity include the conventional method for traditional TTA planning [1], a correction method [2], the Common Tangent method (CT) [11], the Tibial Anatomy-based Method (TAM) [12], the modified TTA planning method [3], the Bielecki method (BM) [13], the osteotomy axis method [14], Margo Cranialis method (MC) [15] and «2,1» method [15] (Table 1).

**Table 1:** Summary of techniques developed to measure required tibial tuberosity advancement for traditional TTA and TTAT and supporting evidence. Methods included in this study are highlighted.

*Technique*	Year	Authors	Further supporting evidence	
*Conventional method*	2002	Montavon et al.	Millet et al. (2013); Pillard et al. (2016)	Conventional method vs. CT Conventional method vs. correction method
***Common Tangent Method***	**2006**	**Dennler et al.**	Millet et al. (2013);	Conventional method vs. CT
*Correction method*	2011	Etchepareborde et al.	Pillard et al. (2016)	Conventional method vs. correction method
***Bielecki method***	**2014**	**Bielecki et al.**	**-**	
***modified TTA planning method***	**2015**	**Kapler et al.**	**-**	
*Tibial Anatomy-based Method*	2016	Ness et al.	Kapler et al. (2015)	modified TTA planning method vs. TAM
*Osteotomy axis method*	2017	Pillard et al.	-	
*Margo Cranialis method*	2022	*Koch et al*.	-	
*2*,*1 method*	2022	*Koch et al*.	-	

Cadmus and colleagues in 2014 compared the use of transparent overlays and imaging software to perform a virtual TTA and proved that the transparent overlay method underestimates the size of the advancement cage required to achieve PTA = 90° [4]. As regards the conventional method, the amount of necessary advancement is measured along a line parallel to the tibial plateau slope [1]. Nevertheless, the displacement of the tibial tuberosity does not follow the same pattern in conventional TTA and new generation TTAT. Since in TTAT the tibial crest is not displaced proximally and advanced in a curvilinear fashion, the methods described for conventional TTA may not be appropriate [16]. For this reason, Etchepareborde et al. described a correction method in which the amount of advancement needed was measured in a cranial direction perpendicular to the tibial mechanical axis [2]. Nevertheless, Pillard and colleagues 2016, comparing the conventional and correction methods, concluded that both radiographic methods do not result in a reduction from PTA to near 90° in 24 canine stifle joints evaluated [16]. Therefore, the currently available literature suggests that transparent overlays, conventional, and correction method have poor reliability for preoperative planning of TTAT [2, 4, 8, 16].

The required TTA could also be determined by the common tangent method described by Dennler et al. [11], which is based on the evidence that the tibial thrust is neutral when the patellar tendon is perpendicular to the tibial plateau [1] and on the assumption that this should be achieved at a stifle angle of 135°, which resemble the mid-stance phase of the gait cycle [11]. However, further studies detected poor reliability of CT, which seems to lead to under correction [4, 8]. In 2016 Ness proposed the Tibial Anatomy-based Method, which differs from other for the reliance on tibial landmarks exclusively [12]. Furthermore, this method does not consider the femorotibial spatial relationship, avoiding the inaccuracy created by tibial subluxation, as suggested by Bielecki et al. [13]. The clinical outcome of most surgical procedures performed on TAM preoperative planning showed a good follow-up, and none of the complications were attributed to over- or under-advancement of the tibial tuberosity [12, 17]. This method was also compared to modified TTA planning method described by Kapler and colleagues (2015), which accounts for anticipated distal translation of the patella after Maquet Modified Procedure (MMP) [3], without statistical difference in wedge sizes recommendation. However, both methods seem to underestimate the size of wedge needed to provide appropriate advancement, and the authors suggest to increase the measured wedge size by 30% to compensate for the amount of underestimation identified. Still, this correction has not been evaluated to establish its impact on radiographic or clinical outcomes [3].

Bielecki method defined the effect of femorotibial subluxation on these measurements by determining how much additional advancement would be required for each millimetre of subluxation, and developed a formula for calculating the required addition to measured TTA for stifles with cranial tibial subluxation [13]. More recently, Pillard and colleagues (2017) developed a new method taking into account the position and length of the osteotomy, the distal translation of the patella, and the cage placement along the osteotomy site. The distance by which the tibial tuberosity should be advanced to reduce the PTA to 90° was measured along a line perpendicular to the planned osteotomy axis. The osteotomy axis method was also compared to true advancement measurements obtained with Modified Maquet Technique in this ex vivo study, resulting in planning measurements that closely matched true advancement measurements after PTA reduction to 90 ± 1° [14]. However, this method has never been clinically tested.

Although advances have been made in preoperative measurement methods, a discrepancy does exist between the desired tibial tuberosity advancement measured preoperatively and the true one surgically achieved [13, 14]. Considering this literature background, the Authors decided to further investigate the reliability of preoperative measurement methods, including CT because it is the most widely used in reviewed literature [18], TAM for its promising results [12] and BM due to the lack of previous comparison study. The aim of this retrospective study was to analyse CT, TAM, and BM to compare interobserver reliability and assess differences in the amount of TTA measured. To the Authors’ knowledge no previous study investigated CT, TAM and BM in terms of comparison and reliability.

## Materials and methods

The preoperative mediolateral (ML) radiographic projection of adult dogs with CCLD underwent to MMP were retrospectively evaluated (Philosophy HF 400, I.P.S. MEDICAL S.r.l.s., Bussolengo, VR, Italy). Radiographs were included if the joint was close to 135° (a range of ± 5° was tolerated). The long axes of the femur and tibia were identified as described by Dennler and colleagues in 2006 [11]. and if the superimposition of the femoral condyle was < 2mm. The radiographs were obtained under general anaesthesia, collimated with a beam centred over the stifle, including the distal third of the femur, intercondylar eminence above the tibial plateau, the entire tibia, and the talocrural joint. All data were measured by a digital radiographic viewing program (Horos, version 3.3.6., horosproject.org). Three observers represented by an expert surgeon (Ob1), a Ph.D. student (Ob2), and an intern student (Ob3) independently and blinded to the assessment of other observers, evaluated and scored the osteoarthritis (OA) degree, measured the PTA, the amount of advancement of the tibial tuberosity and selected the appropriate wedge to achieved it with three different methods (CT, TAM, and BM). Before starting the study, the Ob3 was adequately trained to use the different measurement methods. The radiographic images were submitted to the observers in three different measurement sessions, one for each method, at a distance of one week, and each time randomly archived in a file folder.

The OA degree for each stifle was evaluated and scored, assessing 11 points in the ML view and grading on a 4-grade scale [19–21].

**Fig 1:**
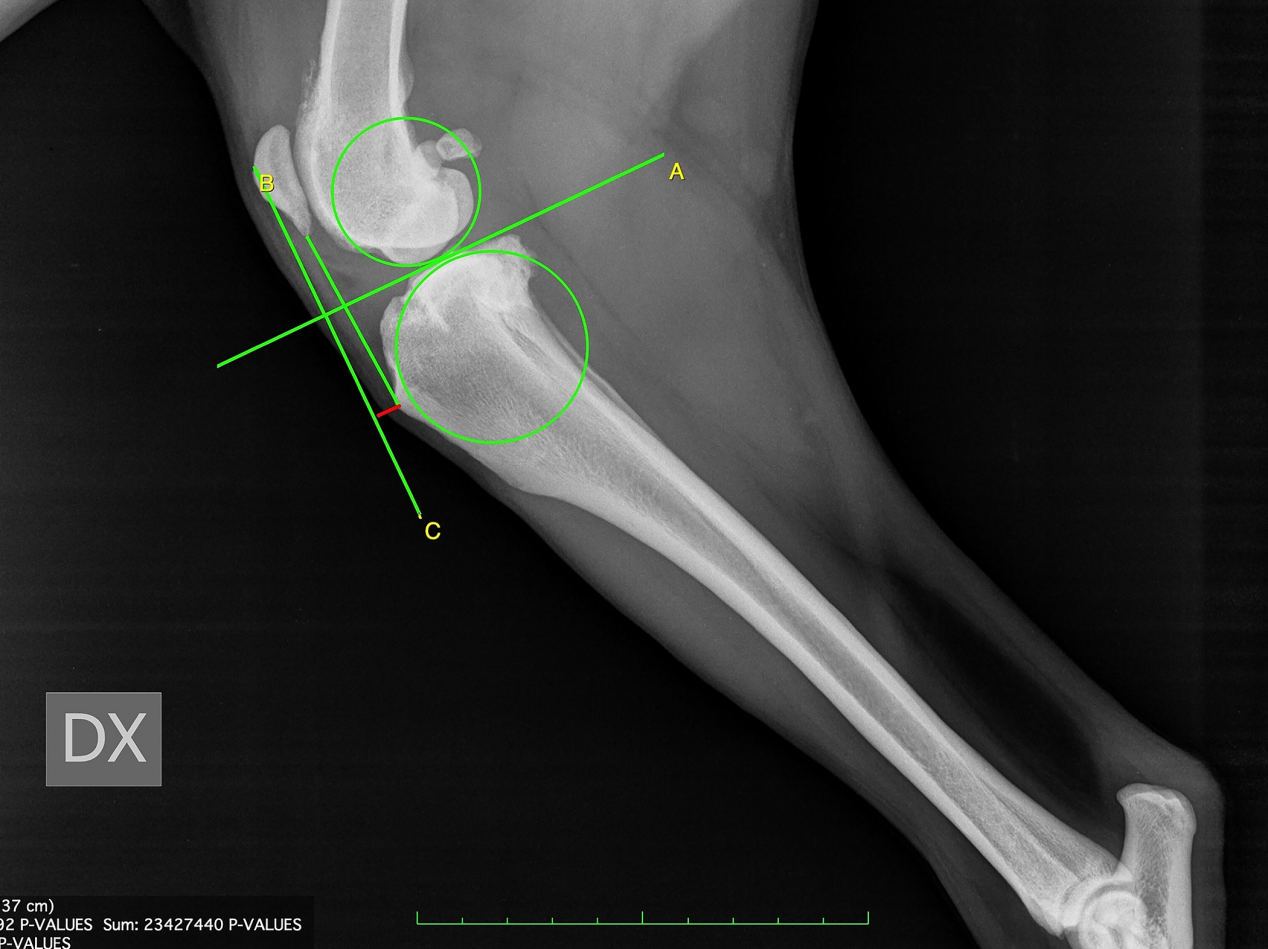
Common tangent (CT) method for the measurement of tibial tuberosity advancement.

**Fig 2:**
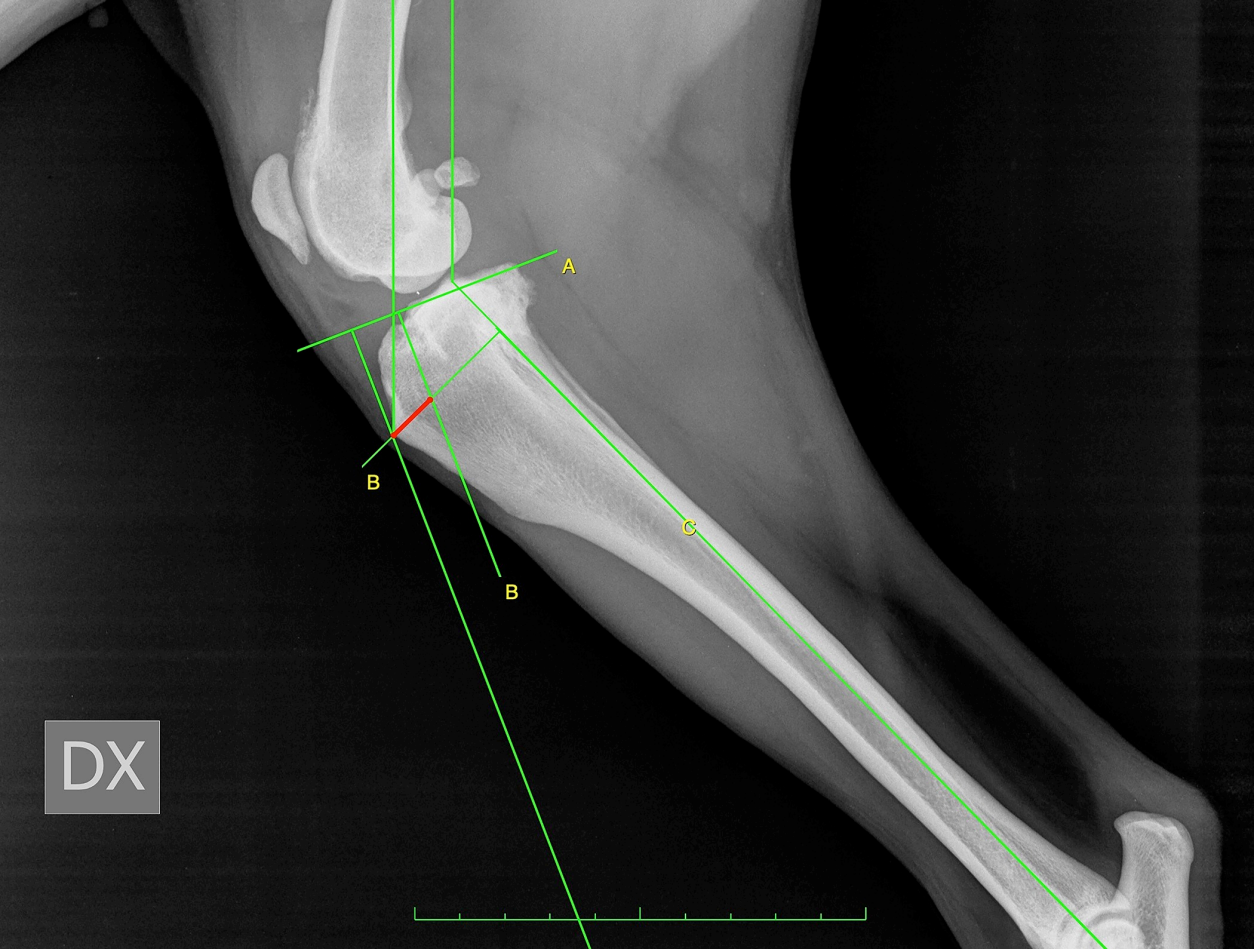
Tibial Anatomy-based Method (TAM) for the measurement of tibial tuberosity advancement.

**Fig 3:**
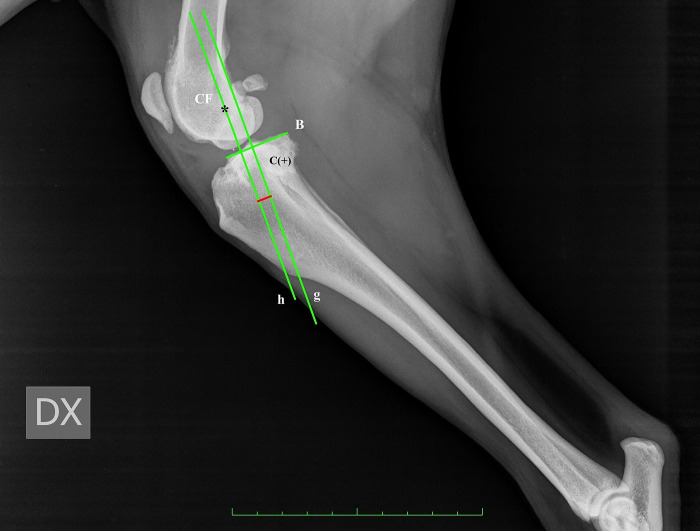
Radiographic landmarks of BM. Center of the femoral condyle (CF), length of the tibial plateau (B), g: line passing through intercondylar eminence, perpendicular to the tibial plateau; h: line passing through CF, perpendicular to the tibial plateau, distance C detect the subluxation value.

Measurement methods were applied as previously described and summarized below:

- For CT, firstly, observers drew two circles representing the femoral and tibial condyles, marking the centre. Next, they connected the two centres with a line and drew the line perpendicular to that, defined as the common tangent. The angle between the common tangent and the line drawn from the caudal margin of the patella to its insertion on the tibial tuberosity corresponded to PTA_CT_. To measure the amount of advancement required, observers considered the distance between the tibial tuberosity and the line perpendicular to the common tangent starting from the cranial margin of the patella [11] (Fig 1).
- For TAM, the tibial functional axis and the tibial plateau were drawn. Secondly, from the functional axis, a caudally directed 135° angle towards the femur was made. Next, a parallel line through the patellar insertion point on the tibial tuberosity was located. This line intersected the tibial plateau line that was previously drawn. A perpendicular line to the tibial plateau was placed starting from patellar insertion. Next, a parallel line through the intersection point was drawn. The distance between this line and the most cranial point of the tibial tuberosity, measured along a line perpendicular to the function axis, was recorded as the request advancement [12] (Fig 2).
- For BM, the advancement measurement was performed by determining a line from the origin of the patellar ligament passing perpendicular to the tibial plateau and calculating the distance from the cranial-most point of the tibial tuberosity to that line. In order to assess the amount of tibial subluxation, the centre of the femoral condyle, intercondylar eminence, and tibial plateau was identified and marked. The length of the tibial plateau (TPL) was recorded. Two lines were drawn perpendicular to the tibial plateau, one passed through the intercondylar eminence, and the second passed through the centre of femoral condyle. The subluxation was calculated as the distance (C) between these two lines. If the line through the centre of the femoral condyle was cranial to the line through the intercondylar eminence, the subluxation amount had a positive value, and vice versa, the value was negative [13] (Fig 3). To define the necessary addition to the measured advancement taking into account the stifle subluxation, we applied the Bieleki formula:

Addiction(mm)=1.091x(TPLx0.201)–C

Where: 1.091 is the additional advancement required for each mm of subluxation, and 0.201 is the mean length in the percentage of the distance between the lines passing through intercondylar eminence and the centre of the femoral condyle, respectively, in intact cruciate ligament stifle.

All data were recorded using spreadsheet software (Microsoft Excel 2019. Microsoft Corporation, Redmond, WA) and imported into a software package (IBM® SPSS® Statistics, Version 26.0. IBM Corporation, Armonk, New York) used to perform the statistical analysis. Continuous variables were presented as mean, standard deviation (± SD), and range. After verifying the data was not normally distributed through the Shapiro-Wilk test, the Kruskal-Wallis test was used to assess the differences of OA scores, the interobserver comparison for each measurement method, and the agreement degree among techniques. Pairwise multiple comparison was used as post hoc test. The significance level for all tests was set equal to 0.05.

The inter-observer reliability for TAM, CT, and BM were assessed using the interclass correlation coefficient (ICC); while the intra-observer reliability for each observer, where the differences among techniques were taken into account, was also performed by ICC. The ICC results were interpreted as follow: poor (ICC< 0.50), moderate (ICC 0.51 to 0.70), good (ICC 0.71 to 0.90), very good (ICC > 0.91).

## Results

Thirty-three stifle radiographs of 24 dogs with CCLD were evaluated. The mean for age and weight were 63.7 ± 24.6 months (range 18–120) and 35.8 ± 8.1 kg (range 19.5–48.5), respectively. Breeds included were mixed breed (n = 8), Labrador Retriever (n = 4), Italian Cane Corso (n = 3), Rottweiler (n = 2), Pittbull (n = 2), Boxer (n = 1), Golden Retriever (n = 1), Bull Mastiff (n = 1), Dogo Argentino (n = 1), Italian Coarsehaired Pointer (n = 1).

Since data were not normally distributed, Kruskal-Wallis test and Pairwise multiple comparison tests were applied. The results, expressed in mean ± SD, are showed in Table 2.

**Table 2:** Pair ways multiple comparison test for preoperative measurement methods and OA degree. Each line tests for the null hypothesis that the distributions for Samples are identical.

		OA			Advancement (mm)		
Observer	Technique	Mean ± SD	Minimum	Maximum	Mean ± SD	Minimum	Maximum
Ob1	CT	18.4 ± 5.4	11	35	10.1 ± 4	0	20
	TAM				9.6 ± 1.7	3	13.5
	BM				12.2 ± 3.6	0	19
Ob2	CT	17.5 ± 4.4	11	35	8.4 ± 3.8	1.2	17.7
	TAM				9.5 ± 2.1	3	15.1
	BM				11.5 ± 5.4	3.4	26.6
Ob3	CT	14.3 ± 2.9	11	22	7.1 ± 3.4	0	13.1
	TAM				10.3 ± 2.2	5.4	16
	BM				11 ± 4.3	1.8	20.4

Interobserver reliability was analysed for each method included. As regards CT, the sample average rank was 10 mm (range 0–20) for Ob1. 7.6 mm (range 1.2–17.7) for Ob2 and 6.32 mm (range 0–13.1) for Ob3. A statistical difference was detected for CT between Ob1 and Ob3 (p <0.001) and between Ob1 and Ob2. No statistical differences between the observers were found for TAM, for which Ob1 obtained a mean value of 10 mm (range 3–13.5), Ob2 of 9.78 mm (range 3–15.1), and Ob3 of 9.98 mm (range 5.4–16). When BM was applied for radiographic assessment, Ob1 got a median of 12.5 mm (range 0–19), Ob2 of 7.6 mm (range 3.4–26.6), and Ob3 of 11.92 mm (range 1.8–20.4). For BM, statistical differences were found between Ob2 and Ob3 (p <00.5) and between Ob1 and Ob2 (p< 0.001).

The intra-observer ICC show a poor agreement for Ob1 (ICC = 0.126), a moderate agreement for Ob2 (ICC = 0.537), and a slight, moderate agreement for Ob3 (ICC = 0.414). Finally, the reliability of the three preoperative planning methods among observers was analyzed by inter-observer ICC and showed a slight moderate agreement for TAM (ICC = 0.731) and poor agreement for CT (ICC = 0.741) and BM (ICC = 0.248).

Mean value among observers for CT was 8.4 mm ± 4, for TAM was 9.9 mm ± 1.9, and for BM, 10.2 mm ± 4.3. The agreement degree among the techniques showed differences between TAM and BM (p< 0.01), CT and BM (p< 0.006), with BM mean rank greater than others.

## Discussion

This prospective study aimed to evaluate three different TTAT preoperative planning methods, CT, TAM, and BM, in terms of comparison and interobserver reliability by three observers with different levels of experience. Moreover, the authors chose to assess the OA degree among the observers to evaluate whether osteophytosis could alter the perception of radiographic landmarks. In our sample, the observer experience seems to affect the overall OA score, as the less experienced observer scored significantly lower compared to the others. This result is not consistent with the literature, where measurement variability between observers was reported to be low [21]. However, our results probably suffered from the low number of observers, and because only the mediolateral projection was used in our study, they are not fully comparable with previous data. Although several studies have found that the presence of osteophytes does not affect the identification of landmarks such as the tibial plateau and intercondylar eminence [8, 22, 23], our results, even though based on only three observers, make that this may be a bias, especially in less experienced surgeons. Future studies upon the influence of OA on measurement for TTAT planning are needed.

The preoperative planning is an essential step that affects the accuracy of the surgical procedure. The primary goal of TTAT preoperative planning is to assess the amount of advancement required to obtain a postoperative PTA of 90°. Several methods have been developed for this purpose, but few studies have investigated the reliability and degree of agreement between the different measurement methods [3, 8, 16]. The interobserver reliability assesses the concordance degree when multiple observers use the same measurement technique in the same measurement set. By selecting observers with different levels of experience, we also aimed to detect the influence of this variable on the measurements. The main objective of this study was to evaluate interobserver reliability among CT, TAM, and BM. Although CT is the most commonly used method for preoperative planning of TTAT [18], our results showed a significant influence of the observers’ experience and poor interobserver reliability consistent with the data previously reported [8].

One reason for this may lie in the rationale of CT, which relies on the spatial relationship between the tibia and femur, making it inherently unreliable [24]. The need to identify femoral and tibial landmarks and the use of specific graphic tools make the method poorly reproducible. In addition, the observers’ different perception of the OA change, the proper position of the stifles at 135°, and the correct superimposition of the femoral condyles and of the tibial plateau represent a critical point.

The experience did not affect the interobserver reliability for TAM, although slight, moderate reliability was found. Despite its extensive use for TTAT, this method has been investigated only in terms of comparison [3, 18]. Our results could be explained by the fact that TAM is based solely on tibial landmarks. As noted by observers of this study, this method is easier to perform compared to CT and BM, even for less experienced surgeons. Considering only tibial landmarks reduces the difficulties associated with identifying femoro-tibial relationship or positioning the limb correctly. In addition, since only two right angles need to be drawn after identifying the anatomical axis and the tibial plateau, the use of dedicated graphic tools is avoided, reducing potential errors.

In the present study, conflicting results arise from measurements obtained when BM is applied, for which no influence of experience came up clearly. To date, the only report on BM application and reliability is its first description, and its inclusion in this study is based on the excellent interobserver correlation reported [13]. On the other hand, the poor interobserver reliability that emerged from our results does not confirm the findings of Bielecki and colleagues [13].

Furthermore, no significant difference was detected between CT and TAM when comparing the results obtained with the different techniques. This finding is supported by the study of Butterworth and colleagues, for which mean values of advancement measured using TAM corresponding closely to the ones obtained with CT by Samoy et al. in a population with similar body weight. However, no statistical analysis of the data of these two studies is available, so it remains only the authors’ consideration [25, 26]. Conversely, BM showed no agreement with the other techniques included in the present study, with a significantly higher mean rank. This may be due to the addition to the measured advancement with the newly developed formula to correct femoro-tibial subluxation. As reported in the aforementioned study, there is a significant decrease in the advancement measure as the degree of subluxation increases [13]. Among the methods considered, only TAM does not rely on the spatial femoro-tibial relationship, and the degree of subluxation should not affect measurements of TTA. Nonetheless, the measurements obtained with TAM were significantly lower than those obtained with BM. This finding supports DeRooster and VanBree’s study, which assumed that any surgical planning method that relies upon the spatial relationship between tibia and femur could be inherently unreliable [24].

The intra-observer reliability could be interpreted as a measure of how an observer deals with the ambiguity of each technique. The poor agreement assessed can be related to the BM values for which the observers’ discordance was detected.

The retrospective design of this study and the low number of observers represents a limit, as well as sample size, and does not account for anatomic variability in different breeds, making not completely conclusive the interpretation of our results.

In conclusion, the accuracy and reliability of the measurement method are mandatory in the TTAT surgical planning step. Its planning, indeed, passes through different measurements before to leads to wedge selection.

These measurements suffer from a lot of variables, such as surgeon experience, mispositioning of the leg, and the alteration of the radiographic landmarks due to the OA. The chance to use a planning method less affected by these variables could reduce the discrepancy between the desired tibial tuberosity advancement and the actual one. Our results show that TAM seems to be the easier method also for less-experienced surgeons, providing a more functionally accurate measurement of advancement. This radiographic planning technique was developed relying exclusively upon the tibial landmark, avoiding the errors related to the femorotibial relationship in terms of positioning and landmarks identification. Unfortunately, it is impossible to precisely preoperatively detect the advancement amount of tibial tuberosity that would determine good clinical recovery after cranial cruciate ligament rupture.

According to our findings, none of the measurement methods investigated has good interobserver and intraobserver reliability. This study, anyway, paves the way to further research on the clinical effects of preoperative planning on true advancement obtained through TTAT.
